# More Links but Fewer Effective Routes: Entropy and Resilience in Global Lithium, Cobalt and Nickel Trade Networks

**DOI:** 10.3390/e28080913

**Published:** 2026-08-14

**Authors:** Guoxu Liu, Dong Mu, Tianyu Li, Mingqian Sun, Liu Chen

**Affiliations:** 1 School of Economics and Management, Beijing Jiaotong University, Beijing 100044, China; lgxbjtu@163.com (G.L.); ltybjtu@163.com (T.L.); mingqian_sun@bjtu.edu.cn (M.S.); 2 College of Urban and Environmental Sciences, Peking University, Beijing 100871, China; chenliusylvia@pku.edu.cn

**Keywords:** critical minerals, information entropy, multilayer networks, supply-chain resilience, temporal network modelling

## Abstract

Counts of trade links are often used as evidence of diversification, yet they say little about how value is distributed across those links. Using bilateral flows for selected lithium, cobalt and nickel products among 72 economies from 2010 to 2024, we built directed, value-weighted networks and examined them with multiscale entropy measures, lagged formation models and disruption tests. Here, entropy is used in the information-theoretic sense to measure how evenly trade value, network weight or motif participation is distributed across routes and structural modes; for route-value entropy, exp(H) is the effective number of equally weighted routes. The number of lithium links increased from 180 to 290, but its entropy-effective route count declined from 38.68 to 12.75. Nickel displayed a similar divergence, falling from 118.84 to 21.81 effective routes as links increased, whereas cobalt moved in the opposite direction. Across layer-years, flow entropy was associated with the share of trade retained under targeted attack (ρ = 0.754; Holm-adjusted *p* = 0.001). Binary dependence between lithium and nickel rose over time, although their weighted divergence was still 0.907 in 2024. In the China-removal experiment at the largest capacity margin, 95.7% of nodes survived but only 33.8% of trade value remained. For these product baskets, a larger network therefore need not be a more diversified one, and preserved connectivity can coexist with substantial economic loss.

## 1. Introduction

The transition from fuel-intensive to material-intensive energy systems is reshaping international dependence. Batteries, electric vehicles and grid storage require large, timely flows of lithium, cobalt and nickel, yet extraction, conversion and refining remain geographically concentrated [1,2,3]. Assessments of mineral criticality therefore combine geological availability, production concentration, substitutability and institutional response [4,5,6]. Aggregate availability is only part of the resulting policy problem. A trade system must also retain independent pathways that can preserve function when a major producer, processor or logistics hub is disrupted.

Edge count, density, centrality and concentration ratios are all used to characterise trade-network diversity, although each describes a different property. Adding many small links can enlarge a network while a limited set of corridors continues to carry most of its value. Information measures focus directly on this distribution. Shannon entropy quantifies uncertainty in a probability distribution [7,8]; exponentiating it gives the number of equally weighted routes that would generate the same entropy. Spectral entropy describes the distribution of connectivity and weight across graph modes [9,10]. Jensen–Shannon divergence compares weighted-flow patterns, whereas mutual information measures dependence in binary ties [11,12]. Read together, these measures help separate functional diversification from the addition of peripheral links around a concentrated core.

Where battery-material bottlenecks arise depends on chemistry, production capacity and the stage of the supply chain at which concentration occurs [1,2,13,14]. Olivetti et al. showed that such bottlenecks can occur at several material and processing stages, not only at extraction [15]. Subsequent work has mapped cobalt trade across life-cycle stages [16] and traced critical nodes and failure propagation in lithium and cobalt supply chains [17,18]. Mineral-specific studies have also examined how cobalt, nickel and lithium networks evolve or withstand disruption [19,20,21,22,23]. Studies of complex networks now draw a clearer distinction between robustness and recovery, with cascade outcomes varying by network structure and shock design [24,25]. Three gaps remain relevant to the present study.

First, most analyses consider one mineral or industrial stage at a time and cannot separate binary layer overlap from similarity in the allocation of trade value. Second, entropy is often linked to resilience without testing whether the association persists after stable differences between mineral layers are removed. Stress tests have begun to combine entropy with loss distributions [26], but evidence from an observed, evolving multilayer mineral system is scarce. Third, explanatory and predictive models are seldom evaluated within the same temporal design. Static centrality or ERGM coefficients may describe structure without showing whether they improve out-of-sample prediction beyond gravity and simple tie persistence.

Here we analyse annual bilateral trade in selected battery-relevant lithium, cobalt and nickel products among 72 economies from 2010 to 2024. The lithium layer covers lithium oxide and hydroxide, and lithium carbonate; it does not represent the complete lithium mining chain. We quantify flow entropy, entropy-effective routes, weighted von Neumann entropy, triad-profile entropy, cross-layer Jensen–Shannon divergence, binary normalised mutual information and country-level layer entropy. We then integrate these measures with a Poisson pseudo-maximum-likelihood gravity model, static ERGM diagnostics, a lagged dyadic network-formation model, rolling-origin prediction, interpretable ordered boosting, a structural resilience index, targeted removals and load-redistribution cascades.

The practical question is straightforward: when more country pairs begin to trade, do buyers gain several meaningful alternatives, or does most value still pass through a few corridors? A second question follows. If two mineral layers use some of the same country pairs, do they also distribute value in the same way? Finally, when a key country is removed, does preserving network connections also preserve trade value?

We answer these questions in four linked stages. The entropy analysis compares nominal network growth with the distribution of trade value and reveals a link-proliferation paradox, with cobalt providing a directional contrast within the selected product baskets. Cross-layer measures then separate shared trading relationships from similarity in the routes that carry value. The formation and prediction models test whether these cross-layer relationships add information beyond the history of each mineral network. Stress tests finally distinguish restoration of connectivity from replacement of the economic function lost with a hub. Diversification is therefore evaluated through meaningful route dispersion rather than partner count alone.

Our contribution is therefore not the claim that conventional network measures are uninformative, but that they answer a different question. Partner count, degree and density measure nominal connectivity; entropy-effective routes measure how many equally weighted routes would generate the observed distribution of economic value [27]. Networks with similar numbers of links can consequently exhibit different levels of effective diversification and vulnerability.

The four analytical components are sequential rather than interchangeable. Complex-network analysis establishes topology and cross-layer roles in the multilayer system [28]. Information-theoretic measures quantify the dispersion of economic value, with exp(H) converting route-value entropy into an effective number [27]. Lagged formation models then evaluate tie persistence and cross-layer dependence while preserving temporal order [29]. Targeted removals and load-redistribution tests finally assess whether observed structures retain connectivity and trade value under attack [30,31]. This sequence connects structure, value allocation, formation and disruption without treating descriptive topology, statistical association and stress-test outcomes as causal equivalents.

## 2. Conceptual Framework and Hypotheses

**Figure 1:**
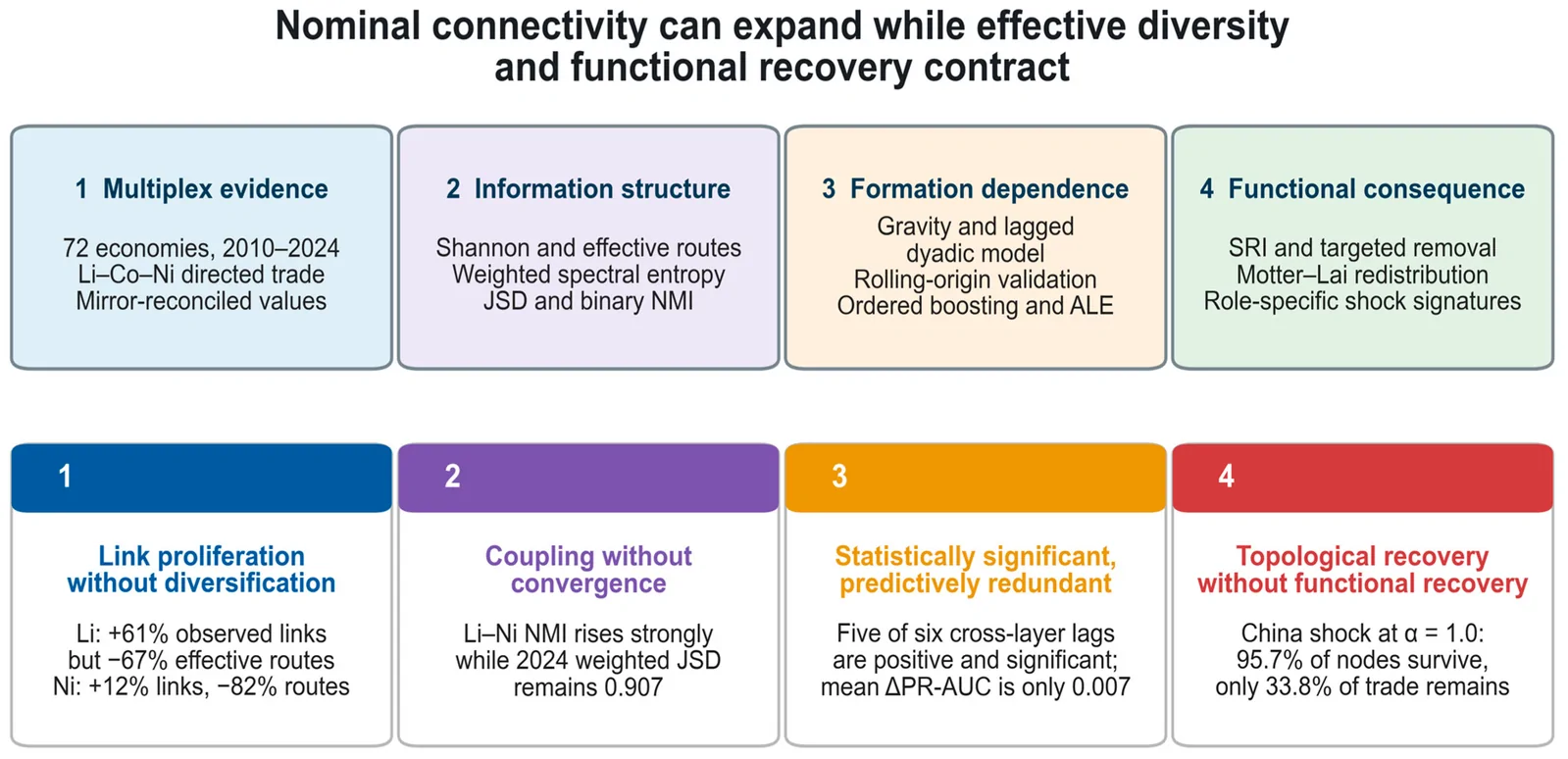
Analytical logic and evidence boundaries. The upper sequence links the multilayer data, information measures, lagged dyadic formation model and stress tests. The lower cards summarise the four separations examined in the manuscript. JSD, Jensen–Shannon divergence; NMI, normalised mutual information; ALE, accumulated local effects; SRI, structural resilience index. Associations are retrospective, the formation model is not a generative TERGM and cascades are stress tests rather than forecasts.

Figure 1 is a guide to the argument rather than an empirical result. Read from left to right, it connects the multilayer trade data to information measures, models of link formation and, finally, disruption experiments. The lower row states the four comparisons tested in the paper. Its main contribution is to keep the interpretation boundaries visible: entropy describes how trade is distributed, temporal models describe conditional association and prediction, and stress tests describe controlled failure scenarios rather than forecasts.

### 2.1. Entropy–Connectivity Decoupling

Within each mineral layer, the value shares of active routes form a probability distribution. An even distribution produces high Shannon entropy and a large effective number of routes; concentration in a few corridors produces low values. Edge count need not follow the same pattern because newly observed links may carry little trade. Normalising entropy by the logarithm of the number of observed routes separates evenness from network size. The effective-to-observed route ratio then records how the value-weighted effective route set compares with nominal connectivity.

**H1.**  *An increase in observed trade links is not sufficient for greater information-theoretic diversity. When added links are predominantly peripheral, flow entropy and the entropy-effective route ratio decline.*

### 2.2. Entropy and Functional Resilience

Route diversity may provide alternatives after disruption, but none of the entropy statistics is itself a measure of resilience. Shannon flow entropy describes value dispersion, weighted von Neumann entropy describes spectral dispersion and triad entropy describes motif heterogeneity. Whether a network withstands disruption also depends on reachability, path length, persistence and spare structural capacity. The SRI contains partner diversity, so its association with flow entropy is convergent rather than independent evidence. Trade retained after targeted removal provides the more direct functional test.

**H2.**  *Within mineral layers, higher trade-flow entropy is positively associated with trade retained under targeted attack. Its relationship with the composite SRI is treated as secondary, convergent evidence.*

### 2.3. Cross-Layer Information Coupling

Layer overlap has both a binary and a weighted form. Binary overlap is present when the same country pairs trade in more than one mineral; weighted similarity additionally requires comparable value shares to pass through those pairs. Shared ports, processors, finance, customers and due-diligence systems may raise binary mutual information. Differences in technology and industrial scale can nevertheless keep Jensen–Shannon divergence high. A single symmetric quantity would therefore miss part of the coupling between layers.

**H3.**  *Binary dependence between mineral layers increases over time, while their weighted flow distributions remain distinguishable. The magnitude and trend of this coupling differ across layer pairs.*

### 2.4. Temporal Network Formation and Prediction

Creating and maintaining a mineral trade tie involves qualification, logistics, contracting and regulatory familiarity. These sunk costs favour persistence, while shared partners can reduce uncertainty and encourage closure. Gravity variables delimit the opportunity set, but the previous network should carry more information about annual link formation. A lag from another mineral layer may still contribute when trade in one material precedes trade in another, although the additional predictive value may be modest.

**H4.**  *Previous-year ties and lagged triadic closure positively predict current links after gravity controls. Within-layer dynamics improve out-of-sample prediction relative to gravity and persistence-only baselines, while the additional gain from multilayer variables is smaller.*

### 2.5. Critical Nodes and Heterogeneous Shock Signatures

A system hub, a layer specialist and a broker expose the network in different ways. Loss of a system hub can affect several layers at once. Removing a layer specialist may eliminate substantial value without fragmenting the topology, whereas removing a broker may reduce reachability despite a modest direct trade share. Random removal supplies a reference distribution; MNCI-ranked removal deliberately directs failure towards structurally important nodes. The relevant comparison is whether these roles yield different combinations of node survival, component survival and retained trade.

**H5.**  *Under identical stress-test assumptions, countries with different multilayer roles produce distinct combinations of topological loss, direct value loss and cascading failure.*

## 3. Materials and Methods

### 3.1. Product Scope, Data Sources and Sample

We assembled annual bilateral trade records from UN Comtrade for 2010–2024 [32]. The balanced network universe contains 72 economies, including the principal producers, processors, consumers and trading hubs in the retrieved panel. Seventy economies reported exports at least once, and all 72 economies appeared either as import reporters or trade partners. Keeping this universe fixed made the network panel computationally tractable, but excluded some peripheral Comtrade reporters. The analysis therefore concerns the covered global core rather than the full reporting universe.

The product basket is listed in Table 1. Its lithium layer contains HS 282520 (lithium oxide and hydroxide) and HS 283691 (lithium carbonate). HS 253090 was excluded because it is a residual mineral category rather than a lithium-specific product, and HS 262090 did not provide a consistent lithium-specific series in the retrieved panel. The cobalt layer contains HS 260500, 282200 and 810520. The nickel layer contains HS 260400, 720260, 750120 and 750210, with HS 282540 and 750110 added only in the expanded-basket robustness test. Time-invariant conversion factors were applied to reported net weights solely for a descriptive audit of approximate contained metal. They were not used in the network weights, entropy measures or formation models. The primary analysis accordingly measures exposure in reconciled current-dollar trade value rather than physical metal mass.

GDP came from the World Bank’s World Development Indicators [33]. Population-weighted bilateral distance, contiguity, common official language and colonial links came from the CEPII GeoDist and Gravity databases [34,35]. Missing GDP values were interpolated only within each economy’s observed time span and were not extrapolated across long gaps. Monetary values are reported in current US dollars. A linear time trend and a 2020–2021 indicator capture common temporal shifts in the formation model, but cannot fully separate prices from quantities. The 2010–2024 window also spans rapid electric-vehicle expansion, the COVID-19 pandemic and heightened geopolitical attention to critical minerals; these developments provide historical context but are not treated as causally identified interventions.

Exporter-reported value X^E^_ijmt_ and its importer-reported mirror X^I^_ijmt_ often differ because of freight valuation, timing, partner attribution and reporting quality. We used their arithmetic mean when both values were positive and retained the available value when only one was reported:

(1) $${\bar{X}}_{ijmt}=\left\{\begin{array}{cc} \frac{X_{ijmt}^{E}+X_{ijmt}^{I}}{2} & \mathrm{if}\;\mathrm{both}\;\mathrm{reports}\;\mathrm{are}\;\mathrm{positive}\\ max\left(X_{ijmt}^{E},X_{ijmt}^{I}\right) & \mathrm{otherwise} \end{array}\right.$$

Here i denotes the exporter, j the importer, m the mineral layer and t the year. Robustness tests replaced the arithmetic mean with exporter priority, importer priority or the geometric mean. We removed self-trade, aggregate “world” partners and observations outside the 72-economy universe before aggregation. A directed edge was present when reconciled annual trade reached at least USD 100,000. Alternative thresholds of USD 10,000 and USD 1 million were evaluated separately.

### 3.2. Multilayer Network Construction and Descriptive Measures

For each year, we represented the trade system as a directed, value-weighted multilayer graph G_t_ = {G_t_^L^, G_t_^c^, G_t_^N^}. The layers share a common node set, and an edge may occur in one or more layers. Binary adjacency and reconciled current-dollar weight are defined as

(2) $$A_{ijt}^{m}=1\left[{\bar{X}}_{ijmt}\geq 10^{5}\right],W_{ijt}^{m}={\bar{X}}_{ijmt}$$

Descriptive measures include active nodes, edge count, density, reciprocity, transitive closure, total trade, degree, strength, betweenness, coreness and layer participation. We used weighted flows to represent economic importance and binary edges to model link formation, thereby limiting the influence of a few very large transactions on topology. Maps display the smallest ranked set of 2024 links that accounts for at least 80% of value within each layer. This common cumulative threshold avoids an arbitrary top-k rule and permits comparison across minerals.

The primary information measure is normalised Shannon entropy of route value [7,8]. For layer m in year t, each active directed route has share p. Raw entropy is the negative probability-weighted log share, and division by ln(M) bounds the index between zero and one:

(3) $$p_{ijmt}=\frac{W_{ijmt}}{\sum_{u,v}W_{uvmt}},H_{mt}^{f}=-\frac{\sum_{i,j}p_{ijmt}ln\left(p_{ijmt}\right)}{ln\left(M_{mt}\right)}$$

We also exponentiated raw entropy to obtain the number of equally weighted routes that would produce the observed uncertainty. Dividing this quantity by the observed edge count gives the entropy-effective route ratio:

(4) $$N_{mt}^{eff}=exp\left(-\sum_{i,j}p_{ijmt}ln\left(p_{ijmt}\right)\right),\eta_{mt}=\frac{N_{mt}^{eff}}{M_{mt}}$$

Two structural measures distinguish flow evenness from topology. We symmetrised each weighted directed network by summing opposite-direction values within each unordered country pair. The combinatorial Laplacian L was normalised to unit trace and treated as a density matrix. The resulting von Neumann entropy was divided by ln(N − 1) [9,10]. We also converted the 16-state directed triad census into a probability distribution and divided its Shannon entropy by ln (16).

(5) $$\rho_{mt}=\frac{L_{mt}}{Tr\left(L_{mt}\right)},H_{mt}^{VN}=-\frac{Tr\left(\rho_{mt}ln\left(\rho_{mt}\right)\right)}{ln\left(N-1\right)}$$

Cross-layer weighted similarity was evaluated over the common vector of all directed dyads. Jensen–Shannon divergence used base-2 logarithms and ranges from zero for identical distributions to one for disjoint distributions [11,12]:

(6) $$\begin{array}{c} \mathrm{JSD}\left(P,Q\right)=\frac{1}{2}D_{KL}\left(P∥M\right)+\frac{1}{2}D_{KL}\left(Q∥M\right)\\ M=\frac{P+Q}{2} \end{array}$$

Binary coupling was measured as normalised mutual information (NMI) between two-layer adjacencies. NMI equals zero under independence and one for identical, non-degenerate binary states:

(7) $$NMI\left(A,B\right)=\frac{I\left(A;B\right)}{\sqrt{H\left(A\right)H\left(B\right)}}$$

At country level, node-layer entropy is the normalised Shannon entropy of total-strength shares across lithium, cobalt and nickel. High values indicate balanced multilayer participation, whereas low values indicate specialisation. We computed all entropy measures on the USD 100,000 network and repeated them at the alternative thresholds.

### 3.3. Gravity Model for Trade Intensity

We estimated the PPML gravity model on dyad–layer–year observations:

(8) $$\begin{array}{c} E\left(V_{ijmt}|X\right)=\exp\left(\beta_{1}z\left(ln{GDP}_{it}\right)+\beta_{2}z\left(ln{GDP}_{jt}\right)\right)\\ +\beta_{3}z\left(ln{DIST}_{ij}\right)+\gamma^{T}Z_{ij}+\mu_{m}+\delta z\left(T_{t}\right) \end{array}$$

Here V_ijmt_ is reconciled trade value, Z_ij_ contains contiguity, common language and colonial links, μ_m_ denotes layer indicators and γT_t_ is a linear time trend. Logged GDP, logged distance and the trend were z-standardised before estimation. Their coefficients therefore represent a one-standard-deviation increase in the logged predictor, not a 1% elasticity. Standard errors were clustered by country pair. PPML retains zero trade and avoids the retransformation bias of log-linear OLS under heteroskedasticity [36].

### 3.4. Static ERGM Diagnostics and Temporal Network Formation

We estimated static ERGM diagnostics separately by layer for reciprocity, geometrically weighted edgewise shared partners (GWESP), sender activity, receiver popularity, GDP and distance. In general form,

(9) $$Pr\left(Y=y|X\right)=\frac{exp\left\{\theta^{T}g\left(y,X\right)\right\}}{\sum_{y^{\prime }\in Y}exp\left\{\theta^{T}g\left(y^{\prime },X\right)\right\}}$$

where g_k_(y, X) are network statistics and θ_k_ are their parameters. These static models describe network structure and are not interpreted as evidence of temporal causation.

The primary formation model conditions current adjacency on the previous year’s network. Full MCMC-based TERGM or bootstrap btergm estimation was not available for the complete panel. We therefore used a lagged dyadic logistic MPLE with ERGM-inspired network statistics and dyad-clustered robust standard errors. This is a temporal network-formation approximation rather than a fully generative TERGM. The reported intervals are not simulation-based measures of TERGM uncertainty. The specification is

(10) $$\begin{array}{c} \mathrm{logitPr}\left(A_{ijt}^{m}=1\right)=\alpha +\beta^{T}X_{ijt}+\rho A_{ij,t-1}^{m}+\eta R_{ij,t-1}^{m}\\ +\lambda {GWESP}_{ij,t-1}^{m}+\sum_{q\neq m}\delta^{q\to m}{CL}_{ij,t-1}^{q\to m}+\psi {MATCH}_{ijm,t-1} \end{array}$$

Dynamic statistics comprise the lagged same-direction edge, lagged reverse edge, lagged GWESP, lagged exporter out-degree and lagged importer in-degree. CL(q → m)_ij,t−1_ equals one when dyad ij traded in source layer q at t − 1 and the outcome is in target layer m. We estimated all six directional cross-layer terms. Position matching is the standardised product of the exporter’s lagged outward-strength percentile and the importer’s lagged inward-strength percentile. The model retained gravity controls, layer indicators, a linear year trend and a 2020–2021 indicator. Coefficients were exponentiated to odds ratios.

Conditional goodness of fit was evaluated on the 2024 holdout using 200 independent Bernoulli draws from the fitted dyadic probabilities. We compared observed and simulated density, reciprocity and transitive closure by layer. These diagnostics assess conditional calibration of the dyadic model; they do not convert it into a generative TERGM.

### 3.5. Rolling-Origin Prediction and Interpretable Boosting

Prediction used strictly temporal splits. For each test year from 2019 to 2024, models were trained on all preceding years; future observations contributed neither features nor scaling information. We compared four specifications: gravity only, persistence only, within-layer dynamics and multilayer dynamics. Performance was measured by ROC-AUC, precision–recall AUC (PR-AUC), Brier score and log loss. We emphasised PR-AUC because active links are sparse. The persistence-only model included the lagged edge and an intercept, providing a stronger comparator than a majority-class baseline.

For the 2024 holdout, we fitted CatBoost models to gravity, within-layer and multilayer feature sets. CatBoost’s ordered target statistics reduce prediction shift in the presence of categorical variables [37]. Hyperparameters were fixed before holdout evaluation. Permutation importance was defined as the reduction in PR-AUC after shuffling a feature. Accumulated local effects (ALE) curves summarised changes across the observed covariate distribution without evaluating implausible combinations of correlated network features [38]. A temporal supra-graph embedding neural network (TSGE-NN) served only as a supplementary robustness benchmark because it did not outperform ordered boosting.

To quantify uncertainty in performance differences, we resampled country-pair blocks 1000 times from the 2024 holdout. For models a and b, we report the percentile interval of ΔPR = PR_a_ − PR_β_. Block resampling preserves within-dyad dependence across layers and is more conservative than resampling individual observations.

### 3.6. Critical Nodes, Structural Resilience and Cascading Stress Tests

We measured system-level criticality with a multilayer node criticality index (MNCI). Nine annual indicators were min–max normalised: strength, PageRank, betweenness, coreness, cross-layer participation, layer scope, direct trade loss, efficiency loss and reachability loss after node removal. Raw CRITIC weights increase with dispersion and decrease with redundancy [39]. To reduce instability, each raw weight was shrunk equally towards 1/J:

(11) $$\begin{array}{c} C_{j}=s_{j}\sum_{k}\left(1-r_{jk}\right),w_{j}^{C}=\frac{C_{j}}{\sum_{h}C_{h}}\\ {\overset{\sim }{w}}_{j}=0.5w_{j}^{C}+\frac{0.5}{J},{MNCI}_{i}=100\sum_{j}{\overset{\sim }{w}}_{j}z_{ij} \end{array}$$

Here, s_j_ is the standard deviation of indicator j, r_jk_ is its correlation with indicator k, J is the number of indicators and the shrinkage coefficient is 0.5. MNCI is the weighted sum of the normalised indicators. We classified nodes as multilayer hubs, brokers or layer specialists according to criticality, participation and betweenness. These labels aid interpretation but do not enter model estimation.

The structural resilience index (SRI) combines six normalised components: partner diversity, defined as one minus the trade-share HHI; global efficiency; the largest weakly connected-component share; year-to-year edge persistence; random-removal robustness; and targeted-removal robustness. The final two components are areas under the corresponding connectivity curves:

(12) $${SRI}_{mt}=100\sum_{j}w_{j}z_{jmt}$$

(13) $$R=\int_{0}^{1}S\left(f\right)df\approx \frac{1}{K}\sum_{k=1}^{K}S\left(\frac{k}{K}\right)$$

Targeted removal followed MNCI rank, whereas random robustness was averaged over repeated random sequences. The targeted–random contrast is therefore a designed benchmark, not an independent test that targeted ordering is superior. Because composite indices depend on weighting, we evaluated equal-weight, entropy-weight and leave-one-indicator-out variants.

The Motter–Lai experiment modelled load redistribution after an initial node loss [30]. Baseline load was unweighted betweenness on the undirected topology of the collapsed 2024 network. Capacity was C_i_ = (1 + α)L_i_^0^, and a node failed when redistributed load exceeded its capacity. We evaluated capacity margins α = 0.2, 0.5 and 1.0. Outcomes were surviving-node share, largest-component share and retained current-dollar trade value. This comparative stress test assumes fixed topology and unweighted shortest-path rerouting. It does not model inventories, price responses, mine expansion, recycling or endogenous partner substitution.

### 3.7. Hypothesis Tests, Robustness and Reproducibility

Within-layer entropy associations and pair-specific temporal trends were tested using 1999 stratified permutations, with Holm correction within each hypothesis family. We also varied the edge threshold to USD 10,000 and USD 1 million, expanded the nickel basket and reconciled mirror flows using exporter priority, importer priority and geometric means. Rolling-origin evaluation was repeated for every year from 2019 to 2024, and the SRI was recomputed under alternative weighting schemes. We compared static and temporal coefficient signs without treating their estimands as equivalent. Finally, we audited a partially observed 2025 file but excluded it from the main analysis because reporter coverage was incomplete.

## 4. Results

The results follow the same sequence as Figure 1. We first establish how the three mineral networks changed, then ask whether additional links created effective alternatives. We next examine why links form and whether the resulting models predict unseen years. The final step identifies critical countries and compares the trade losses produced by different types of node failure.

### 4.1. Network Growth Differs Across Lithium, Cobalt and Nickel

**Figure 2:**
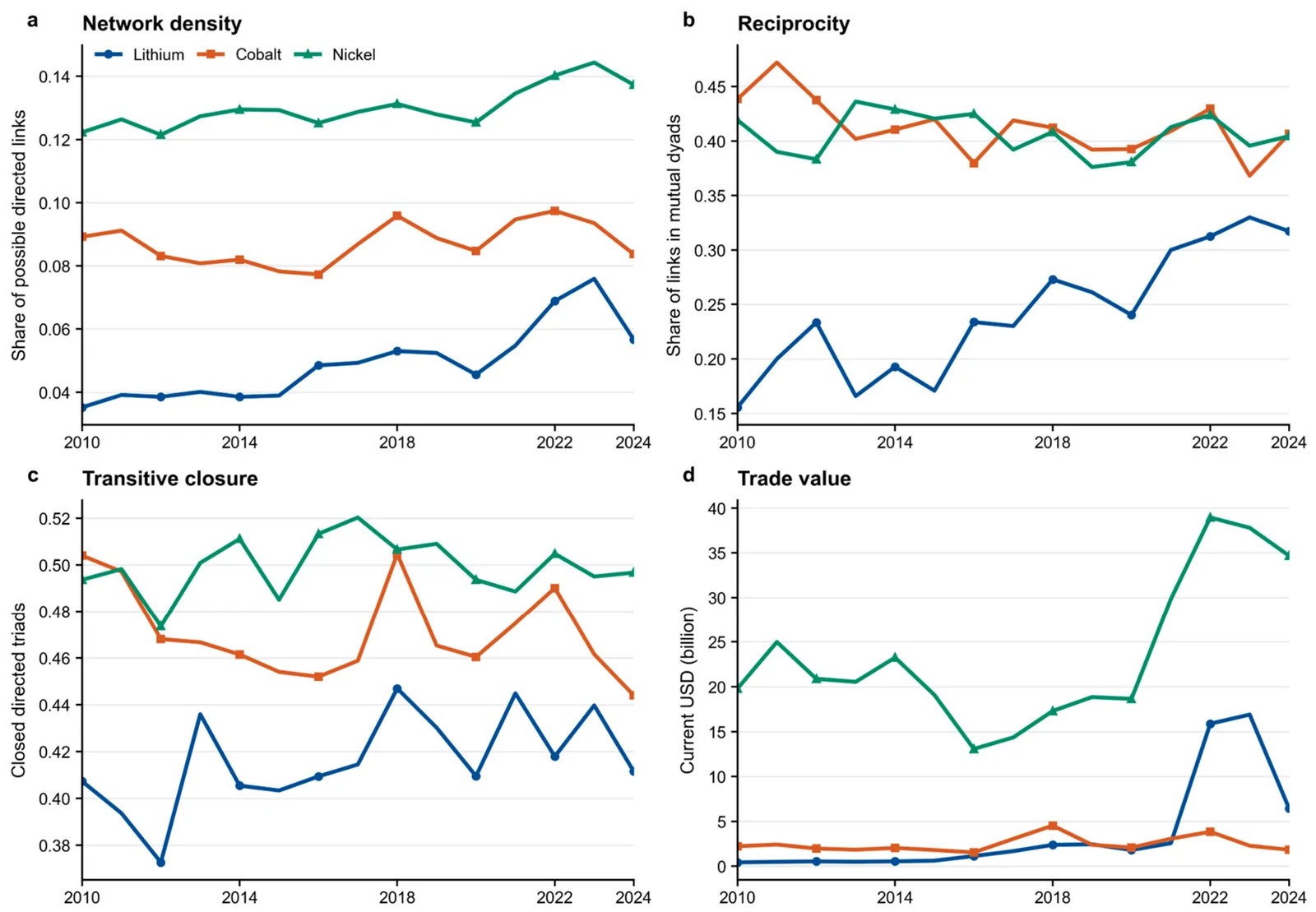
Mineral-specific evolution of the trade layers, 2010–2024. (**a**) Directed density; (**b**) reciprocity; (**c**) transitive closure; and (**d**) mirror-reconciled trade value in current USD. Topology uses the USD 100,000 annual edge threshold.

Figure 2 establishes the empirical baseline. Nickel remains the largest and densest layer, lithium grows most rapidly from a much smaller base and cobalt follows a different and less monotonic path. The four panels also show why no single network statistic is enough: density, reciprocal trading, closure and trade value do not move together in every layer. This figure’s contribution is to show that “network growth” is not one uniform process, which motivates the value-distribution analysis that follows. Endpoint characteristics are reported in Table 2.

The three layers followed distinct trajectories (Figure 2 and Table 2). Lithium recorded the fastest monetary growth. Reconciled trade rose from USD 0.43 billion in 2010 to USD 6.44 billion in 2024. Active nodes increased from 51 to 60, edges from 180 to 290 and reciprocity from 0.156 to 0.317. Over the same period, a growing share of lithium value became concentrated in a small set of routes, and tie persistence weakened towards the end of the sample.

Cobalt began with a comparatively connected network. Between the endpoints, its edge count fell from 456 to 428, trade value from USD 2.23 billion to USD 1.84 billion and density from 8.92% to 8.37%. This decline was not monotonic, and current-dollar totals also reflect prices, product composition and the fixed basket. Nickel remained the largest and densest layer. Edges increased from 625 to 702, active nodes from 62 to 69 and trade value from USD 19.80 billion to USD 34.67 billion. Its 2024 density was 13.73%, more than twice that of lithium.

**Figure 3:**
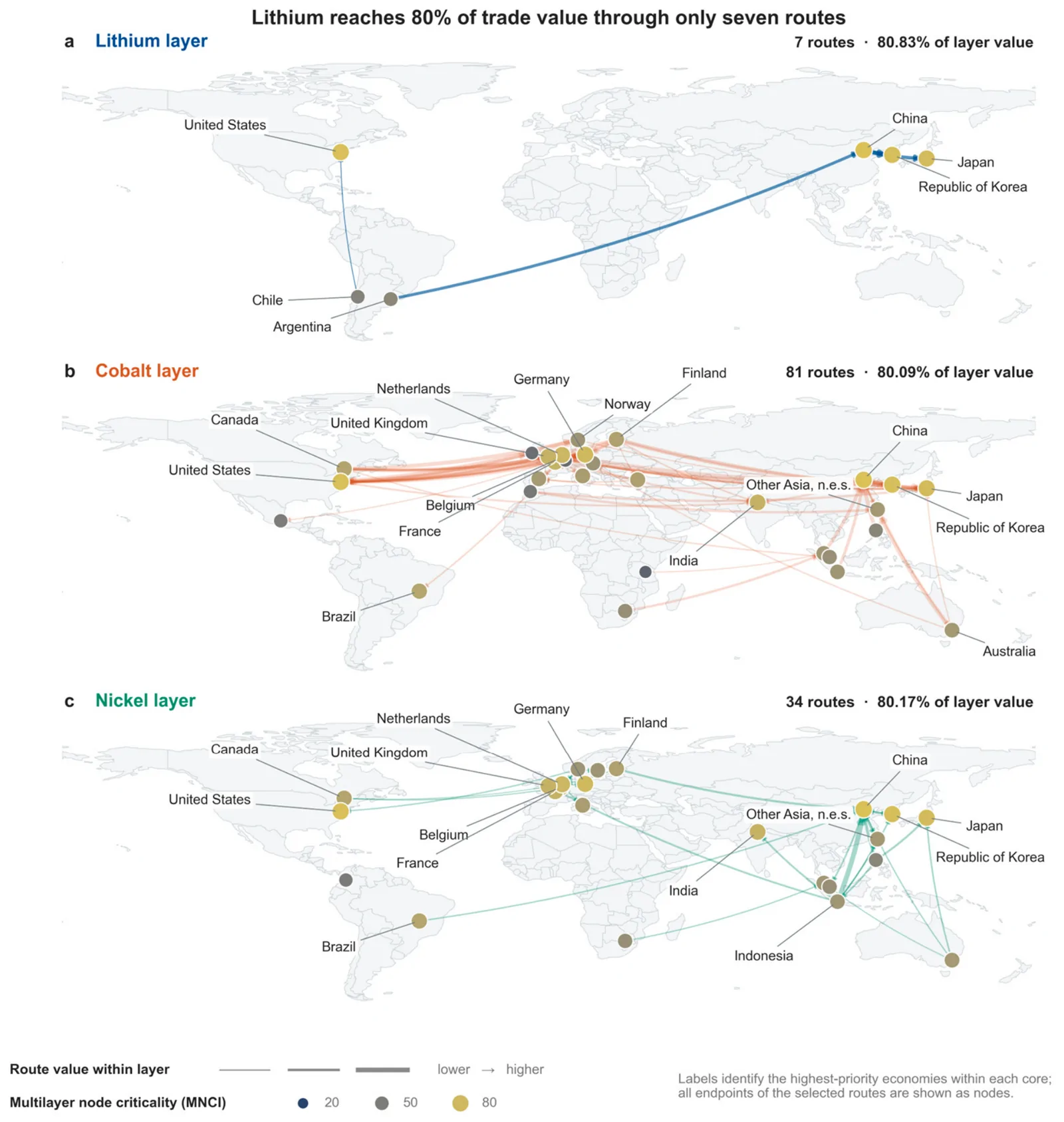
Contrasting spatial footprints of the 2024 80% value cores. Panels (**a**–**c**) show directed lithium, cobalt and nickel routes ranked by reconciled value until cumulative coverage first exceeds 80%. The lithium core contains 7 routes (80.83%), compared with 81 cobalt routes (80.09%) and 34 nickel routes (80.17%). Route width is scaled within each layer, and arrowheads indicate trade direction. Node area and colour encode the multilayer node criticality index (MNCI) on a common 0–100 scale. Labels identify the highest-priority economies within each core; all endpoints of the selected routes are plotted. The maps describe the selected product basket, and antimeridian-crossing routes are omitted only from display. Blue, orange and green route colours denote lithium, cobalt and nickel, respectively; node colour follows the shared MNCI scale.

The 2024 map showed a pronounced difference in value concentration (Figure 3). Seven routes were sufficient to carry more than 80% of lithium trade value. The corresponding counts were 34 for nickel and 81 for cobalt. This contrast matters because the lithium layer added many edges but remained dependent on a small value-carrying core. Nickel’s high-value trade was spread across a wider set of corridors centred on Asian processing and consumption, while cobalt’s 80% core extended over more routes and all three selected product stages.

Figure 3 turns an abstract concentration measure into a geographic picture. The lithium map reaches its 80% value threshold with only seven corridors, whereas cobalt and nickel require many more. Thick lines identify the routes that carry most trade, so a visually sparse core signals dependence even when many thinner relationships exist outside the map. Node labels locate the highest-priority economies, while the common MNCI scale separates geographic participation from system-wide criticality. The figure’s contribution is therefore twofold: it shows where the economically important routes are located and explains why a longer partner list can give a misleading impression of diversification.

### 4.2. More Observed Links Do Not Necessarily Create More Effective Routes

The preceding subsection showed that lithium and nickel added trading relationships. The next question is whether those added links carried enough value to become meaningful alternatives. Entropy-effective routes answer this question by translating an uneven flow distribution into the equivalent number of equally weighted routes.

**Figure 4:**
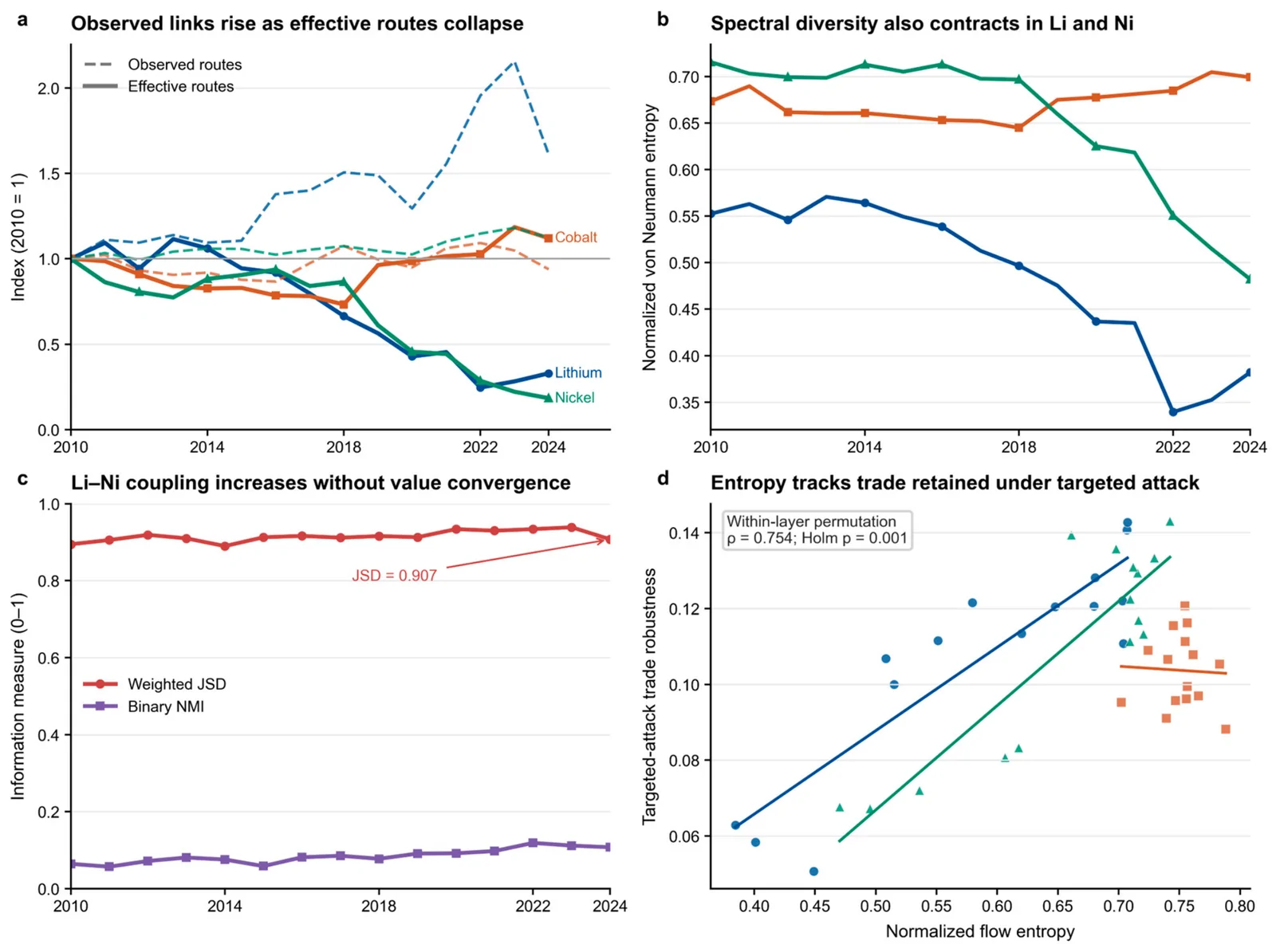
Entropy–resilience decoupling. (**a**) Observed (dashed) and Shannon-effective (solid) routes indexed to 2010; (**b**) normalised weighted von Neumann entropy; (**c**) lithium–nickel JSD and NMI; and (**d**) flow entropy versus targeted-attack trade retention (45 layer-years; stratified permutation ρ = 0.754, Holm-adjusted *p* = 0.001).

Figure 4 contains the central empirical result. Panel (a) shows observed links rising while effective routes contract in lithium and nickel; panel (b) confirms that their weighted network structures also become less diverse. Panel (c) shows that lithium and nickel increasingly share trading dyads without converging in the allocation of value. Panel (d) connects higher flow entropy with greater trade retention under targeted attack. The figure’s contribution is to link network growth, value concentration, cross-layer dependence and resilience in one visual sequence. Endpoint information measures are reported in Table 3.

The information measures supported H1 and separated nominal connectivity from value-weighted effective diversity. Lithium edges increased from 180 in 2010 to 290 in 2024, whereas normalised flow entropy fell from 0.704 to 0.449. The effective number of routes declined from 38.68 to 12.75, and the effective-route ratio from 0.215 to 0.044. Nickel showed the same pattern at larger scale. Its edge count rose from 625 to 702, while flow entropy fell from 0.742 to 0.470 and effective routes from 118.84 to 21.81. Within its selected product basket, cobalt moved in the opposite direction. Observed routes declined from 456 to 428, but flow entropy increased from 0.761 to 0.788 and effective routes from 105.79 to 118.50.

The endpoint direction was invariant to the edge threshold. At USD 10,000, USD 100,000 and USD 1 million, lithium’s 2024 effective-route count was 30%, 33% and 50% of its 2010 value, respectively. The corresponding nickel ratios were 18%, 18% and 18%. Cobalt’s 2024 effective-route count remained 12–13% above its 2010 value at all three thresholds. The main USD 100,000 cutoff therefore did not generate the entropy–connectivity result.

Spectral entropy showed the same endpoint pattern. Normalised weighted von Neumann entropy fell from 0.553 to 0.382 for lithium and from 0.716 to 0.482 for nickel, but rose from 0.673 to 0.699 for cobalt. Triad-profile entropy changed less. The larger shifts therefore occurred in route weights and spectral modes rather than in the range of motif types.

Within-layer permutation tests supported the primary part of H2. After removing stable between-mineral differences, flow entropy was positively associated with trade retained under targeted attack (ρ = 0.754, Holm-adjusted *p* = 0.001). It was also associated with the SRI (ρ = 0.513, adjusted *p* = 0.0025). The latter is convergent evidence rather than independent validation because partner diversity contributes to the SRI. Neither association identifies a causal effect of entropy on resilience.

Cross-layer results partly supported H3 and distinguished topological overlap from weighted similarity. Lithium–nickel binary NMI increased from 0.065 in 2010 to 0.108 in 2024, with a strong positive trend (ρ = 0.911, Holm-adjusted *p* < 0.001). Its Jensen–Shannon divergence nevertheless increased from 0.895 to 0.907. Lithium–cobalt NMI rose from 0.113 to 0.153, while divergence remained close to 0.8. Cobalt–nickel had the greatest binary overlap and lowest weighted divergence, but neither series showed a Holm-significant trend.

### 4.3. Economic Size, Distance and Static Network Structure

The entropy results establish what changed in the distribution of trade. Before interpreting network mechanisms, we separate those patterns from the familiar gravity forces that make trade more likely between large economies and less likely over long distances. The corresponding PPML estimates are reported in Table 4.

The PPML estimates established the gravity baseline. A one-standard-deviation increase in logged exporter GDP multiplied expected trade by 2.237 (coefficient, 0.805). The corresponding multiplier for logged importer GDP was 5.586 (coefficient, 1.720). A one-standard-deviation increase in logged distance multiplied expected trade by 0.571 (coefficient, −0.560). All three estimates differed from zero at *p* < 0.001. Contiguity was negative at the 10% level after adjustment for the remaining controls, whereas common language and colonial history were not significant. These are standardised conditional associations, not raw elasticities.

Static ERGM diagnostics yielded positive GWESP and degree–related coefficients in all layers. Reciprocity was positive and significant for cobalt and nickel, but not for lithium. Because static ERGMs combine formation and persistence, we treated these coefficients as structural descriptions rather than temporal tests.

### 4.4. Past Trading Relationships Dominate New Link Formation

Gravity explains the broad opportunity to trade, but it does not show how strongly an existing relationship carries into the next year. The temporal model therefore adds the previous network, closure and cross-layer pathways to the gravity controls.

**Figure 5:**
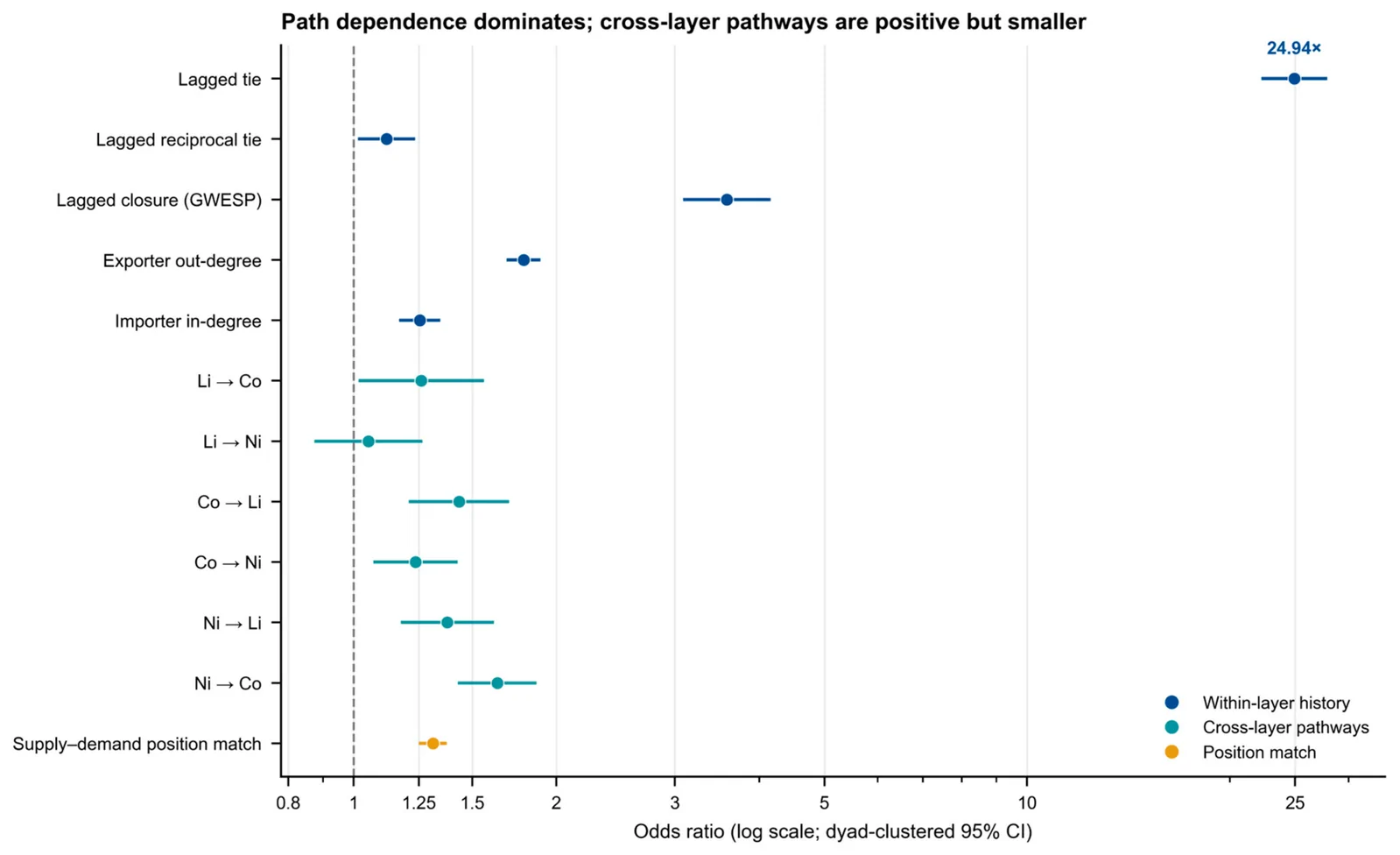
Lagged multilayer network-formation odds ratios. Points and bars show conditional odds ratios and dyad-clustered 95% confidence intervals from the dyadic logistic MPLE specification on a logarithmic scale. The intervals are not MCMC- or bootstrap-based generative TERGM intervals.

Figure 5 makes the hierarchy of formation mechanisms visible. The previous-year tie lies far to the right of every other estimate, showing that persistence is the dominant predictor of a current link. Shared-partner closure and several cross-layer pathways are positive but much smaller. The figure’s contribution is to distinguish a statistically detectable cross-layer effect from the much stronger inertia of established trade relationships. The corresponding coefficients are reported in Table 5.

The lagged formation model supported the path-dependence component of H4. The lagged same-direction edge coefficient was 3.216 (robust SE, 0.055), equivalent to an odds ratio of 24.94. This was the largest coefficient in the model. The lagged reverse-edge coefficient was smaller but positive (0.113, *p* = 0.017). Lagged GWESP was 1.277, corresponding to an odds ratio of 3.58 for the standardised closure statistic. Exporter out-degree and importer in-degree were also positive.

Five of the six directional cross-layer coefficients were positive and significant. Nickel-to-cobalt was largest (0.491; odds ratio, 1.63), followed by cobalt-to-lithium (0.360; 1.43), nickel-to-lithium (0.320; 1.38), lithium-to-cobalt (0.231; 1.26) and cobalt-to-nickel (0.212; 1.24). Lithium-to-nickel was small and non-significant (0.051). Position matching was positive (0.271; odds ratio, 1.31), consistent with greater tie probability when exporter capacity and importer demand occupied similar positions.

Coefficient directions were stable under alternative thresholds and the expanded nickel basket. Under exporter-priority, importer-priority and geometric-mean mirror reconciliation, 2024 edge-set Jaccard similarity with the main network ranged from 0.941 to 0.962. The lagged-edge coefficient remained between 3.049 and 3.140. Strong persistence, positive closure and a weak lithium-to-nickel pathway were therefore robust to these specifications.

Conditional simulations reproduced transitive closure in all three 2024 layers and reciprocity in lithium and cobalt. They overpredicted density in every layer. Observed versus mean simulated density was 0.0599 versus 0.0906 for lithium, 0.0861 versus 0.0995 for cobalt and 0.1409 versus 0.1564 for nickel. Nickel reciprocity was also slightly below the simulated 95% interval. The model therefore ranked future ties well but did not fully reproduce aggregate network sparsity.

### 4.5. Most Predictive Information Comes from Within-Layer History

A significant coefficient does not necessarily improve prediction for a future year. We therefore move from explanation to a stricter test: models are trained only on earlier years and evaluated on the next unseen year.

**Figure 6:**
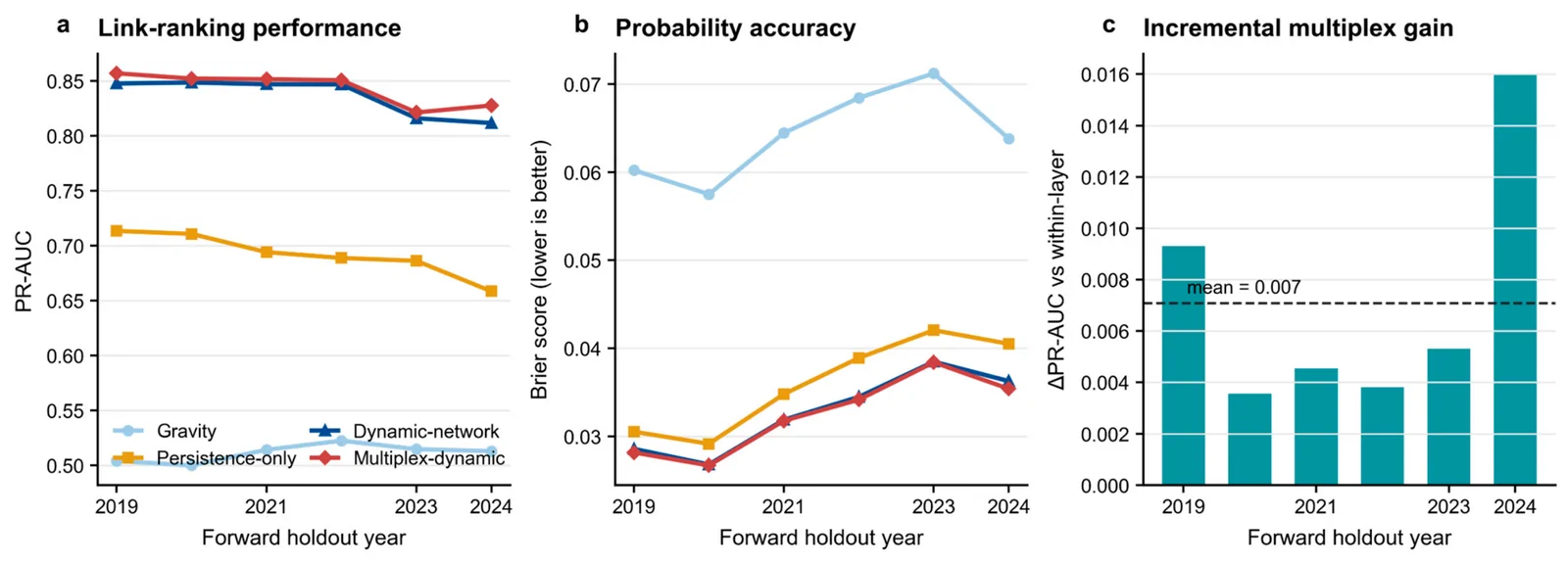
Rolling-origin link prediction, 2019–2024. (**a**) PR-AUC; (**b**) Brier score, for which lower values indicate better probabilistic accuracy; and (**c**) yearly PR-AUC gain of the multilayer dynamic model over the within-layer dynamic model. Each test year is predicted using only preceding years.

Figure 6 shows that network history adds substantial predictive value beyond gravity, but that the within-layer and multilayer dynamic models remain close to one another. Their ranking is stable across the rolling test years, while the small gap between the two dynamic curves represents the limited incremental gain from cross-layer variables. The figure’s contribution is to show that multilayer dependence is real but largely redundant once each mineral’s own history is known. Mean rolling-origin performance is reported in Table 6.

Rolling-origin results supported H4, with an important qualification. Across 2019–2024, the gravity model achieved a mean ROC-AUC of 0.874 and PR-AUC of 0.511. Persistence alone increased PR-AUC to 0.692. The within-layer and multilayer dynamic models reached 0.836 and 0.843, respectively. Brier scores decreased from 0.064 for gravity to 0.036 for persistence and 0.033 for both dynamic models.

The ranking was unchanged in every test year. Multilayer PR-AUC ranged from 0.857 in 2019 to 0.828 in 2024 and was slightly higher than within-layer PR-AUC each year. The mean increment was approximately 0.007. Performance declined modestly in 2023–2024, but the model hierarchy did not change.

**Figure 7:**
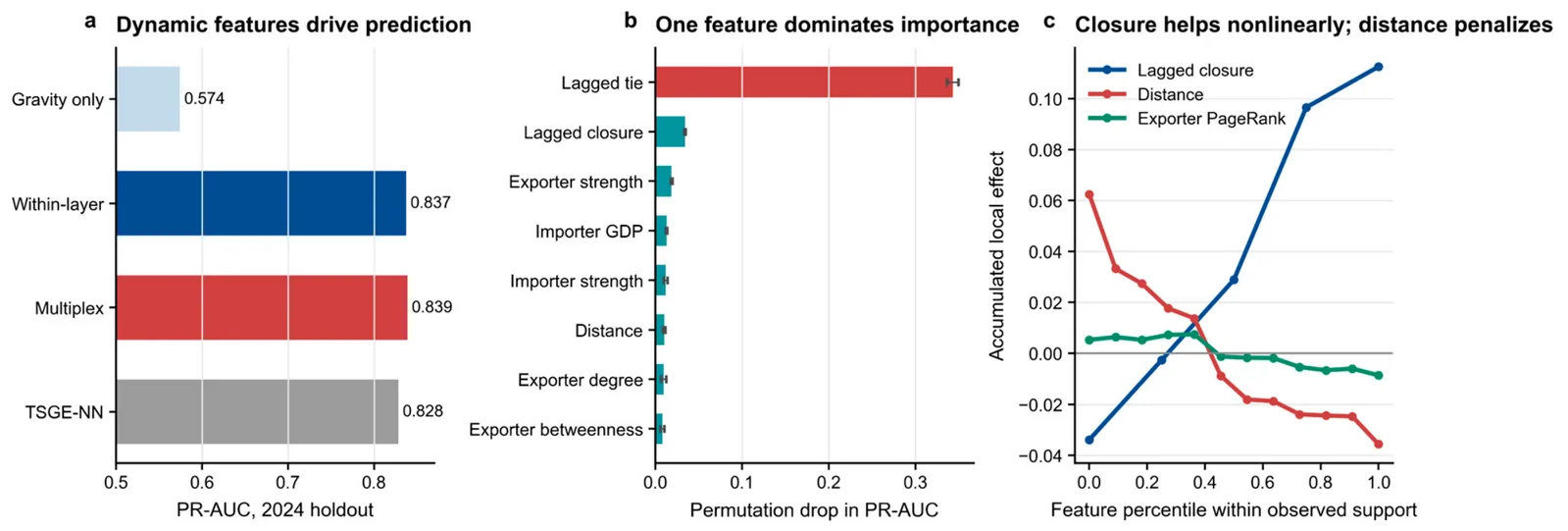
Ordered-boosting performance and nonlinear interpretation for the 2024 holdout. (**a**) PR-AUC across gravity, within-layer, multilayer and neural specifications. (**b**) Permutation importance measured as the reduction in PR-AUC after feature shuffling; error bars are standard deviations across repeated permutations. (**c**) Accumulated local effects over feature percentiles within observed covariate support. Importance and ALE are predictive summaries, not causal effects. In panel (**b**), red highlights the dominant lagged-tie feature, whereas teal denotes the remaining predictors.

Figure 7 explains where the predictive performance comes from. The lagged tie dominates permutation importance, closure becomes increasingly helpful in the upper part of its observed range and distance has a negative contribution. The neural benchmark does not improve on ordered boosting. The figure’s contribution is to connect model performance with an interpretable feature hierarchy while keeping the causal boundary clear: importance and ALE describe prediction, not mechanism. The corresponding 2024 metrics are reported in Table 7.

The 2024 ordered-boosting comparison produced the same hierarchy. Gravity-only CatBoost achieved PR-AUC 0.574, the within-layer feature set 0.837 and the multilayer set 0.839. In country-pair block bootstrap resampling, the within-layer gain over gravity was 0.263 (95% interval, 0.236–0.291). The multilayer-minus-within-layer gain was 0.00165 (95% interval, −0.00155–0.00518). The TSGE-NN benchmark reached PR-AUC 0.828 and did not outperform ordered boosting.

Permutation importance identified the lagged edge as the dominant feature: shuffling it reduced PR-AUC by 0.343. Lagged GWESP ranked second (0.034), followed by exporter out-strength (0.019), importer GDP (0.013), importer in-strength (0.012) and distance (0.010). ALE curves changed sharply when the lagged-edge indicator moved from zero to one. They also showed an accelerating positive contribution of closure in the upper part of its observed range.

### 4.6. Critical Countries Produce Different Kinds of Loss

Prediction addresses which links are likely to appear; resilience asks what happens when an important country disappears. We therefore turn from dyads to country roles and compare system hubs, brokers and layer specialists under the same stress-test assumptions. The 2024 criticality ranking is reported in Table 8.

The 2024 MNCI ranked China, the United States, Korea, Japan, Belgium and Germany as the six most critical system-level nodes. The United States, Korea, Japan, Belgium and Germany participated broadly across layers. China combined exceptional direct-loss exposure with a more concentrated layer profile. The Netherlands and India were prominent specialists. High MNCI scores therefore reflected different combinations of participation, brokerage and flow dominance.

**Figure 8:**
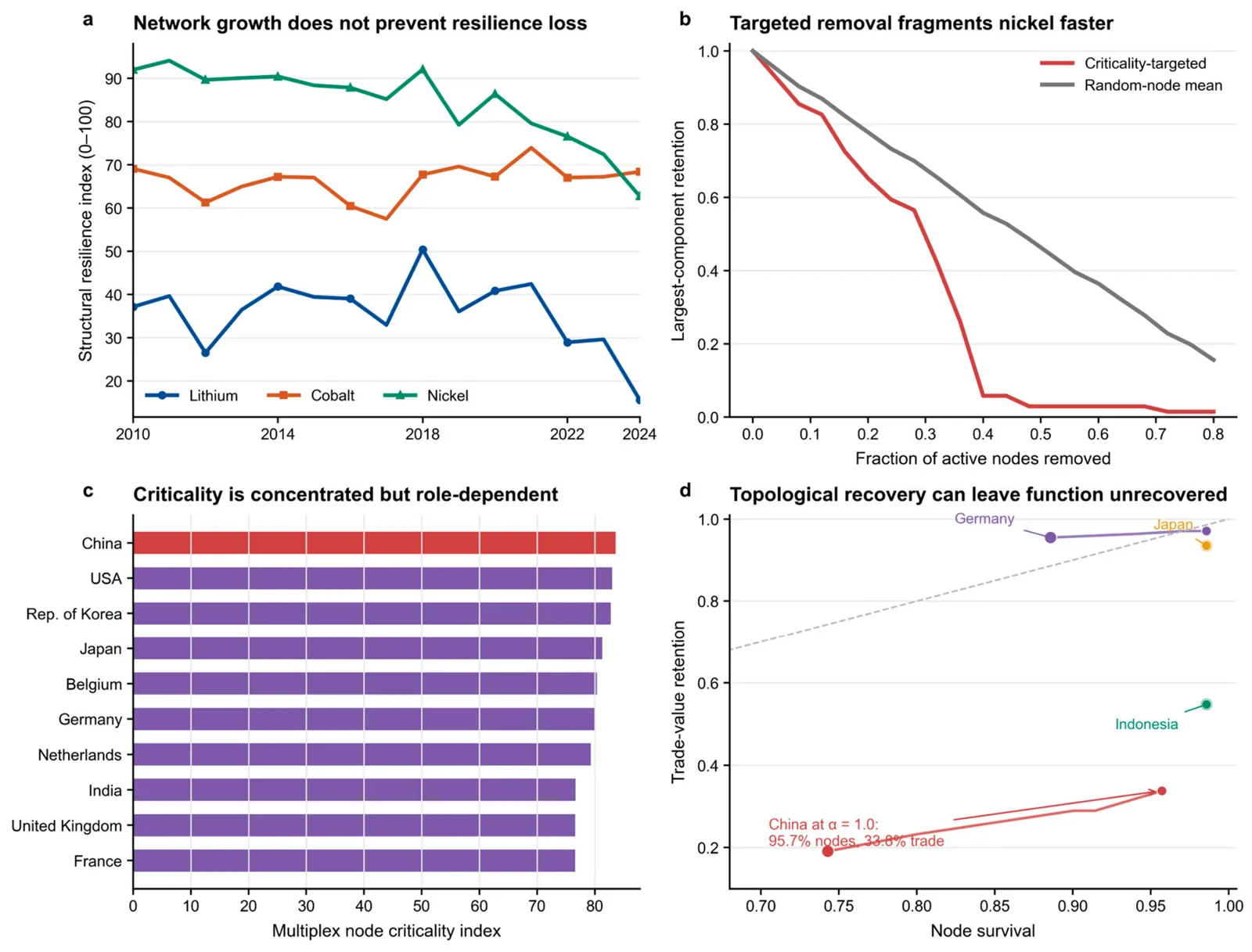
Structural resilience and role-specific shock signatures. (**a**) Layer-level SRI; (**b**) largest-component retention under targeted and random nickel removals in 2024; (**c**) highest 2024 multilayer criticality scores; and (**d**) node survival versus trade-value retention across Motter–Lai capacity margins α = 0.2–1.0. The diagonal denotes equal topological and trade retention. Cascades hold demand and the initial network fixed and are stress tests rather than forecasts. In panel (**c**), red highlights China and purple denotes the other ranked economies.

Figure 8 brings the resilience evidence together. The time series show declining resilience for lithium and nickel; targeted removal fragments networks faster than random removal; the criticality panel shows that leading countries occupy different roles; and the final panel separates node survival from retained trade after a hub shock. Its main contribution is to demonstrate that a network can remain largely connected while losing much of its economic value, so topological recovery and functional recovery must be reported separately.

The SRI declined from 37.14 to 15.57 for lithium and from 91.96 to 62.69 for nickel between 2010 and 2024. Cobalt changed only slightly, from 69.08 to 68.39, despite an intermediate trough. Alternative weights preserved the broad ordering. Spearman correlations between CRITIC rankings and equal-weight or entropy-weight rankings were 0.998 and 0.975, respectively.

MNCI-ranked removal reduced connectivity faster than the random benchmark in every layer, as expected from a targeting rule constructed to prioritise critical nodes [40,41]. The role-specific stress tests addressed H5. At a capacity margin of 0.2, removing China left 74.3% of nodes and 70.0% of the largest component, but only 19.1% of trade value. Increasing the margin to 1.0 raised node survival to 95.7%, whereas retained trade reached only 33.8%. Removing Indonesia left 98.6% of nodes connected but retained 54.7% of trade. Germany’s removal produced greater secondary failure, with 88.6% node survival, but retained 95.5% of value. Japan’s multilayer shock preserved 98.6% of nodes and 93.5% of value. These distinct signatures supported H5.

## 5. Discussion

The results can be read through three contrasts. The number of observed links is compared with the number of effective value-carrying routes; binary overlap is compared with similarity in weighted flows; and topological recovery is compared with retained trade. These contrasts provide the bridge from the statistical results to their supply-chain interpretation.

### 5.1. Why More Links Can Still Mean Less Diversification

Measured in links, both lithium and nickel appear to have diversified. The value-weighted evidence points in the other direction: each layer gained bilateral ties while losing flow entropy, effective routes and weighted spectral entropy. In 2024, lithium had 290 observed links but only 12.75 entropy-effective routes; nickel had 702 links but 21.81 effective routes. Many of the additional ties therefore contributed little to the dispersion of observed trade value. A partner-count dashboard would have recorded diversification even as value became more concentrated.

Cobalt did not follow this pattern within the selected basket. Its edge count fell, but its effective-route count and principal entropy measures increased. The comparison across layers is important: entropy did not move mechanically with edge count. Instead, the three trajectories show that relational breadth can expand while value-weighted route diversity narrows. Flow entropy was more strongly associated with targeted trade retention (ρ = 0.754) than with the composite SRI (ρ = 0.513). Part of the SRI association is structural because partner diversity enters the index. Entropy is therefore informative about one dimension of resilience, not resilience as a whole [24,26].

### 5.2. Layers Can Overlap Without Carrying Value in the Same Way

The cross-layer results point to a different mismatch. Binary NMI between lithium and nickel increased, but their Jensen–Shannon divergence was still 0.907 in 2024. In practical terms, the same country pair can trade both minerals while carrying only a marginal share of value in one layer. Shared infrastructure or market access can therefore increase binary overlap without making the two value distributions functionally substitutable. The layers became more coupled, but they did not converge.

The predictive evidence is more qualified. Five of the six directional cross-layer lags were positive and significant, yet multilayer variables increased mean PR-AUC by only 0.007 beyond the within-layer model. For the 2024 CatBoost comparison, the bootstrap interval for that increment included zero. Much of the predictive signal shared across minerals was already present in same-layer persistence and closure. Cross-layer terms can still describe conditional dependence and may be useful in scenario analysis, but their statistical significance does not by itself establish a mechanism or demonstrate predictive necessity.

One coefficient warrants separate interpretation. The lagged-tie odds ratio was 24.94, which is consistent with relational capital accumulated through qualification, contracting, logistics and regulatory familiarity. The estimate is not causal: unobserved contracts, policy and firm investment may also generate persistence. Ordered boosting and ALE recovered the same predictive hierarchy, although feature importance cannot identify the underlying mechanism.

### 5.3. A Network Can Stay Connected While Losing Most Trade

The China-removal experiment makes the difference between topological and economic recovery particularly clear. Raising the capacity margin from α = 0.2 to α = 1.0 increased node survival from 74.3% to 95.7%, whereas retained trade rose only from 19.1% to 33.8%. Spare topological capacity prevented many secondary failures, but it could not reproduce the economic function lost with the initial hub. Node survival and largest-component size can consequently overstate functional recovery.

The country cases clarify why node role matters. Removing Indonesia produced a layer-specialist volume shock: 98.6% of nodes survived, but only 54.7% of trade remained. Germany caused more secondary failures at α = 0.2 while preserving 95.5% of value, a pattern consistent with brokerage loss. Japan preserved both topology and most value. A single centrality ranking cannot translate these different signatures into a single intervention [24]. System hubs, layer specialists and brokers should be evaluated separately, although these stress tests do not identify the cost or effectiveness of possible policy responses.

Lithium is the clearest case for caution in monitoring. Trade value grew rapidly, yet the effective-route ratio fell to 0.044 and the SRI to 15.57. Additional low-value partners do not establish that processing, transport or contractual capacity can be substituted. Those functions would have to be measured directly before an observed route could count as evidence of physical supply diversification.

### 5.4. Limitations

The findings are bounded first by product and country coverage. The lithium layer represents compounds rather than the full ore-to-battery chain, and the included product stages are not matched across minerals. Peripheral reporters also fall outside the 72-economy sample. Because current-dollar weights combine changes in prices, quantities and product composition, the entropy measures cannot be read as physical-mass diversification. Country-level trade records further omit corporate ownership and foreign direct investment, both of which can reallocate apparent national exposure in lithium, cobalt and nickel supply [3]. The estimates consequently remain conditional on the product basket, mirror reconciliation, country selection and edge threshold. A stage-matched analysis would require physical quantities, mine output, refinery ownership, inventories, firm contracts and transport capacity.

The temporal-model results require a separate caution. Estimation used dyadic logistic MPLE with lagged network statistics, not MCMC or bootstrap TERGM. Conditional simulations overpredicted 2024 density in every layer and slightly overpredicted nickel reciprocity; good rolling-origin discrimination does not remove that aggregate misfit. The model should not be treated as a fully generative TERGM unless it is replicated with “tergm” or “btergm” and evaluated with simulation-based degree, shared-partner and triad diagnostics [42,43].

Evidence linking entropy to resilience is also limited to retrospective association. The layer-year sample contains 45 observations. Permutation inference is preferable to asymptotic claims for this sample, but it cannot identify causality. Nor is the SRI independent of value dispersion, because partner diversity is one of its components. Weighted von Neumann entropy was calculated from a symmetrised projection and thus discarded direction. Directed Laplacians, Rényi entropies, temporal entropy rates and maximum-entropy multilayer null models are possible extensions.

Node-removal and Motter–Lai experiments should be interpreted as stress tests, not forecasts. MNCI-ranked removal concentrates failure on critical nodes by construction, making its contrast with random removal descriptive. The cascade model fixes demand and the initial network, omits price-mediated rerouting and redistributes load along unweighted shortest paths. More behavioural estimates of resilience would require agent-based substitution, explicit capacity constraints, recovery time and policy-event scenarios.

Taken together, these limitations define the study’s inferential boundary: the networks measure value-weighted trade exposure rather than complete physical substitutability, the models identify conditional association and predictive structure rather than causal mechanisms, and the disruption experiments compare controlled stress scenarios rather than realised recovery paths.

## 6. Conclusions

Across the selected battery-material product baskets, additional trade links did not consistently produce a more even distribution of value or stronger performance under stress. The combined entropy, network-formation, prediction and disruption evidence supports three empirical comparisons that link counts alone would miss.

Lithium and nickel gained ties while their effective route sets and spectral diversity contracted; cobalt moved in the opposite direction within its selected basket. Binary overlap also proved different from weighted convergence. Lithium–nickel dependence increased, yet the weighted flow distributions remained highly divergent and significant cross-layer lags added little predictive information beyond within-layer history. The stress tests produced the third comparison. After removal of China, high spare capacity restored 95.7% node survival but retained only 33.8% of trade value.

Monitoring based on supplier counts alone would miss these differences. Observed links need to be read alongside entropy-effective routes, weighted spectral entropy, cross-layer divergence and role-specific stress losses. Evidence on processing, logistics, ownership, contracts and inventories is still required before a trade route can be treated as physically substitutable. Within the stated product and stress-test boundaries, the framework identifies cases in which connectivity grows while value dispersion or recovery remains limited.

Future research can extend the framework in four directions. First, stage-matched physical-quantity networks should connect mines, refineries, precursor and cathode production, and battery manufacturing with ownership, contract, inventory, logistics and processing-capacity data. Second, fully generative temporal models with simulation-based goodness-of-fit tests could examine whether the estimated formation mechanisms reproduce degree, shared-partner and triad distributions. Third, directed Laplacians, Rényi entropies, temporal entropy rates and maximum-entropy multilayer null models could extend the information-theoretic analysis. Fourth, behavioural disruption models should incorporate price-mediated substitution, capacity-constrained rerouting, demand adjustment and recovery time. Event-study or quasi-experimental designs could then distinguish long-run network evolution from responses to the COVID-19 pandemic, industrial policy and geopolitical shocks.

## Figures and Tables

**Table 1:** Product basket and contained-metal conversion factors.

Layer	HS Code	Product	Factor	Status
Lithium	282520	Lithium oxide and hydroxide	16.5% LCE	Main basket
Lithium	283691	Lithium carbonate	18.8% LCE	Main basket
Cobalt	260500	Cobalt ores and concentrates	1.0% Co	Main basket
Cobalt	282200	Cobalt oxides/hydroxides; commercial cobalt oxides	30.0% Co	Main basket
Cobalt	810520	Cobalt mattes/intermediates; unwrought cobalt and powders	99.5% Co	Main basket
Nickel	260400	Nickel ores and concentrates	1.5% Ni	Main basket
Nickel	720260	Ferronickel	15.0% Ni	Main basket
Nickel	750120	Nickel oxide sinters and other intermediates	35.0% Ni	Main basket
Nickel	750210	Unwrought non-alloy nickel	99.8% Ni	Main basket
Nickel	282540; 750110	Nickel oxides/hydroxides; nickel mattes	65%; 50% Ni	Expanded-basket check

Note: Factors were applied only to reported net weights in a descriptive contained-metal audit. Main network weights, entropy measures and formation models use reconciled current-dollar trade value. The expanded nickel basket is used only in robustness analysis.

**Table 2:** Endpoint characteristics of the three trade layers.

Layer	Year	Active Nodes	Edges	Density (%)	Reciprocity	Closure	Trade (USD bn)
Lithium	2010	51	180	3.52	0.156	0.407	0.43
Lithium	2024	60	290	5.67	0.317	0.412	6.44
Cobalt	2010	60	456	8.92	0.439	0.504	2.23
Cobalt	2024	58	428	8.37	0.407	0.444	1.84
Nickel	2010	62	625	12.23	0.419	0.494	19.80
Nickel	2024	69	702	13.73	0.405	0.497	34.67

Note: Binary topology uses the USD 100,000 threshold; values are mirror-reconciled current US dollars.

**Table 3:** Endpoint information measures and structural resilience.

Layer	Year	Observed Routes	Flow Entropy	Effective Routes	Effective/Observed	von Neumann Entropy	SRI
Lithium	2010	180	0.704	38.68	0.215	0.553	37.14
Lithium	2024	290	0.449	12.75	0.044	0.382	15.57
Cobalt	2010	456	0.761	105.79	0.232	0.673	69.08
Cobalt	2024	428	0.788	118.50	0.277	0.699	68.39
Nickel	2010	625	0.742	118.84	0.190	0.716	91.96
Nickel	2024	702	0.470	21.81	0.031	0.482	62.69

Note: Flow entropy is normalised by ln(observed routes). Effective routes equal exp(raw Shannon entropy). The weighted von Neumann entropy is normalised by ln(N − 1). SRI denotes the structural resilience index.

**Table 4:** PPML gravity estimates for bilateral trade value.

Variable	Coefficient	Cluster-Robust SE	IRR	*p*-Value
ln exporter GDP	0.805 ***	0.095	2.237	<0.001
ln importer GDP	1.720 ***	0.203	5.586	<0.001
ln distance	−0.560 ***	0.137	0.571	<0.001
Contiguity	−0.781 *	0.452	0.458	0.084
Common official language	0.226	0.318	1.254	0.476
Colonial link	−0.327	0.351	0.721	0.352
Time trend	0.074	0.103	1.077	0.472
Cobalt layer	−0.433	0.357	0.649	0.225
Nickel layer	1.827 ***	0.448	6.215	<0.001

Note: Logged GDP, logged distance and the linear time trend were z-standardised. Continuous-variable coefficients and IRRs therefore refer to a one-standard-deviation increase in the logged predictor. Standard errors are clustered by country pair. *** *p* < 0.01; * *p* < 0.10.

**Table 5:** Lagged multilayer network-formation estimates.

Mechanism	Coefficient	Robust SE	Odds Ratio	*p*-Value
Lagged same-direction edge	3.216 ***	0.055	24.936	<0.001
Lagged reverse edge	0.113 **	0.047	1.119	0.017
Lagged GWESP	1.277 ***	0.074	3.584	<0.001
Lagged exporter out-degree	0.581 ***	0.027	1.788	<0.001
Lagged importer in-degree	0.226 ***	0.033	1.254	<0.001
Lithium → cobalt	0.231 **	0.107	1.260	0.030
Lithium → nickel	0.051	0.092	1.052	0.580
Cobalt → lithium	0.360 ***	0.085	1.434	<0.001
Cobalt → nickel	0.212 ***	0.071	1.236	0.003
Nickel → lithium	0.320 ***	0.078	1.378	<0.001
Nickel → cobalt	0.491 ***	0.066	1.634	<0.001
Supply–demand position match	0.271 ***	0.022	1.311	<0.001

Note: Dyad-clustered robust standard errors. Gravity controls, layer indicators, a linear year trend and a 2020–2021 indicator are included. Intervals are MPLE-based, not MCMC/bootstrap TERGM intervals. *** *p* < 0.01; ** *p* < 0.05.

**Table 6:** Rolling-origin out-of-sample performance, mean over 2019–2024.

Model	ROC-AUC	PR-AUC	Brier Score
Gravity	0.874	0.511	0.0643
Persistence only	0.903	0.692	0.0360
Within-layer dynamic	0.969	0.836	0.0327
Multiplex dynamic	0.971	0.843	0.0324

Note: Each year is predicted using only preceding observations. Higher AUC and lower Brier scores indicate better performance.

**Table 7:** Ordered-boosting and neural-model performance for the 2024 holdout.

Model/Comparison	ROC-AUC	PR-AUC or ΔPR-AUC	Brier/95% CI
CatBoost–gravityy	0.900	0.574	0.139
CatBoost–within-layerr	0.971	0.837	0.081
CatBoost–multiplexx	0.971	0.839	0.078
TSGE-NN robustnesss	0.970	0.828	0.076
Within-layer − gravityy	—	0.263	[0.236, 0.291]
Multiplex − within-layerr	—	0.00165	[−0.00155, 0.00518]

Note: Confidence intervals use 1000 country-pair block-bootstrap samples. TSGE-NN is a supplementary robustness benchmark, not the principal model.

**Table 8:** Highest-ranked multilayer critical nodes in 2024.

Rank	Economy	MNCI	Role	Direct Trade-Loss Share	Cross-Layer Participation
1	China	83.61	Layer-dominant system hub	0.648	0.464
2	United States	83.02	Multiplex hub	0.060	0.736
3	Korea	82.74	Multiplex hub	0.086	0.770
4	Japan	81.31	Multiplex hub	0.065	0.829
5	Belgium	80.35	Multiplex hub	0.022	0.789
6	Germany	80.08	Multiplex/bridge hub	0.029	0.612
7	Netherlands	79.29	Layer specialist	0.063	0.405
8	India	76.66	Layer specialist	0.025	0.267
9	United Kingdom	76.62	Layer specialist	0.031	0.463
10	France	76.58	Layer specialist	0.019	0.442

Note: MNCI combines nine normalised topology, participation and removal-loss indicators. Raw CRITIC weights are shrunk 50% towards equal weights. Role labels are descriptive.

## Data Availability

The data presented in this study are available on request from the corresponding author. The underlying bilateral trade records were obtained from the UN Comtrade database, macroeconomic indicators from the World Bank, and bilateral geographic variables from CEPII.
